# Emerging Advances in Combinatorial Treatments of Epigenetically Altered Pediatric High-Grade H3K27M Gliomas

**DOI:** 10.3389/fgene.2021.742561

**Published:** 2021-09-27

**Authors:** Katarzyna B. Leszczynska, Chinchu Jayaprakash, Bozena Kaminska, Jakub Mieczkowski

**Affiliations:** ^1^Laboratory of Molecular Neurobiology, Nencki Institute of Experimental Biology of the Polish Academy of Sciences, Warsaw, Poland; ^2^3P-Medicine Laboratory, Medical University of Gdańsk, Gdańsk, Poland

**Keywords:** pediatric high-grade gliomas, H3K27M, DIPG, combinatorial treatments, epigenetic therapy

## Abstract

Somatic mutations in histone encoding genes result in gross alterations in the epigenetic landscape. Diffuse intrinsic pontine glioma (DIPG) is a pediatric high-grade glioma (pHGG) and one of the most challenging cancers to treat, with only 1% surviving for 5 years. Due to the location in the brainstem, DIPGs are difficult to resect and rapidly turn into a fatal disease. Over 80% of DIPGs confer mutations in genes coding for histone 3 variants (H3.3 or H3.1/H3.2), with lysine to methionine substitution at position 27 (H3K27M). This results in a global decrease in H3K27 trimethylation, increased H3K27 acetylation, and widespread oncogenic changes in gene expression. Epigenetic modifying drugs emerge as promising candidates to treat DIPG, with histone deacetylase (HDAC) inhibitors taking the lead in preclinical and clinical studies. However, some data show the evolving resistance of DIPGs to the most studied HDAC inhibitor panobinostat and highlight the need to further investigate its mechanism of action. A new forceful line of research explores the simultaneous use of multiple inhibitors that could target epigenetically induced changes in DIPG chromatin and enhance the anticancer response of single agents. In this review, we summarize the therapeutic approaches against H3K27M-expressing pHGGs focused on targeting epigenetic dysregulation and highlight promising combinatorial drug treatments. We assessed the effectiveness of the epigenetic drugs that are already in clinical trials in pHGGs. The constantly expanding understanding of the epigenetic vulnerabilities of H3K27M-expressing pHGGs provides new tumor-specific targets, opens new possibilities of therapy, and gives hope to find a cure for this deadly disease.

## Introduction

Pediatric high-grade gliomas (pHGGs) are the prominent group of brain tumors that cause fatalities in childhood (Mendez et al., 2020). Diffuse midline gliomas (DMGs) that arise in midline structures of the brain (such as the brainstem, thalamus, or spinal cord) are the most malignant tumors among pHGGs, and diffuse intrinsic pontine glioma (DIPG) is the most lethal, with only 1% of patients surviving 5 years post-diagnosis (Buczkowicz et al., 2014; Louis et al., 2016). Localization of DIPG to the pons (a major part of the brainstem, above the medulla and below the midbrain) presents a particular challenge for therapy, preventing resection, limiting treatment to radiation with adjuvant temozolomide, and offering only palliative care (Bartels et al., 2011; Jansen et al., 2012; Mendez et al., 2020). The stereotactic biopsies or postmortem tumor samples had been used to derive cell lines and patient-derived xenografts, which proved critical in advancing the drug screens and mechanistic research on the disease (Lin and Monje, 2017; Aziz-Bose and Monje, 2019). Despite recent advancements in understanding the genetic hallmarks of DIPG (Mackay et al., 2017; Filbin et al., 2018; Gröbner et al., 2018), further research into cellular responses to specific drug treatments and therapy resistance is necessary to improve the outcome of patients.

The typical molecular feature of pHGGs is the somatic heterozygous mutation in the *H3F3A* gene encoding the histone H3.3 resulting in the substitution of lysine to methionine at amino acid position 27 of the histone H3 variants (H3K27M), and less commonly in *HIST1H3B/C* and *HIST2H3C*, encoding histones H3.1 and H3.2, respectively (Khuong-Quang et al., 2012; Schwartzentruber et al., 2012; Wu et al., 2012). In the midline pHGGs, the H3K27M substitution occurs in 80% of the cases and is a driver event responsible for the tumor initiation and progression (Pathania et al., 2017; Harutyunyan et al., 2019; Larson et al., 2019; Silveira et al., 2019). It is still debatable whether DIPG starts already during embryonic development or postnatally in early childhood. Numerous experimental studies support the hypothesis that oligodendroglial progenitor-like cells (OPCs) are the cells of origin of DIPG (Monje et al., 2011; Funato et al., 2014; Pathania et al., 2017; Filbin et al., 2018; Nagaraja et al., 2019). In addition, super-enhancers in H3K27M pHGGs had been identified, which reflect the OPC cell of origin (Monje et al., 2011; Nagaraja et al., 2017). Some of the super-enhancers acting in DIPG include genes coding for Ephrins and Ephrin receptors. Receptor tyrosine kinases activated by binding to glycosylphosphatidylinositol (GPI)-linked and transmembrane ephrin ligands may play a role in the diffusive and invasive spread of DIPG through the brainstem (Nagaraja et al., 2017). However, a recent study using inducible pluripotent stem cells with the expression of H3.3-K27M targeted to the endogenous histone locus showed that H3.3-K27M drives tumorigenesis from neural stem cells but not glial progenitors (Haag et al., 2021).

The unique consequence of the H3K27M substitution in pHGGs is a global epigenetic dysregulation, including a loss of H3K27me3 (a repressive mark) and an increase in H3K27 acetylation (an activating mark) at the regulatory region of developmentally regulated genes. These global epigenetic changes occur despite the fact that the protein levels of the H3K27M mutant constitute only 3–17% of the total levels of all H3 histone proteins (Lewis et al., 2013). Since identifying the H3K27M mutation in pHGGs, extensive efforts have been made to characterize the properties of chromatin in these tumors, and druggable targets have started to emerge. In this review, we explicitly focus on the recent advances in epigenetic therapies against H3K27M-bearing pHGGs and especially on the potential of combinatorial therapies targeting chromatin via epigenetic pathways.

## Epigenetic Alterations in H3K27M pHGGs

### Methylation of Lysine 27 in Histone H3

The drop in H3K27me3 in the H3K27M-expressing cells is very striking and consistent in multiple cellular models (Chan et al., 2013; Lewis et al., 2013; Krug et al., 2019; Silveira et al., 2019). The mechanism of the H3K27me3 loss, although still disputable, is mainly attributed to the inhibition of the *N*-methyltransferase enhancer of zeste homolog 2 (EZH2)—the catalytic subunit of PRC2 (polycomb repressive complexes 2). Deposition of the H3K27me3 mark by the PRC2 complex is critical in silencing the expression of specific genes, which, depending on the context, may include tumor suppressors or oncogenes (Chammas et al., 2020). Several studies showed that the H3K27M substitution has a dominant inhibitory effect on the PRC2 complex, which is being preferentially recruited to the H3K27M-containing nucleosomes and retained in an inactive form (Chan et al., 2013; Lewis et al., 2013; Justin et al., 2016; Fang et al., 2018; Diehl et al., 2019). Another study has shown that PRC2 is preferentially recruited to its strongest binding sites in H3K27M-expressing cells, which still retain partial H3K27me3 (Mohammad et al., 2017). However, other studies demonstrated temporary PRC2 recruitment to H3K27M-containing nucleosomes or even exclusion of PRC2 from the H3K27M nucleosomes (Piunti et al., 2017; Stafford et al., 2018). Piunti and colleagues showed that a residual activity of the PRC2 complex is retained outside of the heterotypic H3K27M-H3K27ac nucleosomes and is required for the proliferative potential of the H3K27M-bearing cells (Piunti et al., 2017). Stafford and colleagues, in turn, showed that the H3K27M protein needs to be expressed in great excess compared to the amount of the PRC2 complex in order to inhibit the catalytic activity of PRC2 (Stafford et al., 2018). Furthermore, while PRC2 is only temporarily recruited to H3K27M-containing chromatin, the inhibitory effect on PRC2 is retained even after its release from the chromatin (Stafford et al., 2018). Finally, recent studies by Harutyunyan and colleagues confirmed that PRC2 is indeed recruited to the chromatin in H3K27M pHGGs but is unable to spread the methylation mark from large unmethylated CpG islands (CGIs), which are its high affinity recruitment sites (Harutyunyan et al., 2019). Only a residual amount of H3K27me3 is retained at these CGIs. In addition, they showed that the H3K27me2 mark is deposited outside of CGIs in H3K27M cells and localizes to the areas normally marked by H3K27me3 in H3 wild-type cells (Harutyunyan et al., 2019, 2020).

### Acetylation of Lysine 27 in Histone H3

In addition to decreasing H3K27me3, the H3K27M oncohistone leads to elevated levels of the acetylated H3K27, which is typically associated with active transcription (Wang et al., 2009; Lewis et al., 2013). Some studies showed that the histone H3.3K27M forms heterotypic nucleosomes with the wild-type H3.3, particularly with its acetylated form (Piunti et al., 2017; Krug et al., 2019; Nagaraja et al., 2019). Furthermore, H3K27M-H3K27ac heterotypic nucleosomes recruit bromodomain-containing proteins (BRDs), which are known to recruit RNA Polymerase II (RNA Pol II) and therefore activate transcription (Jonkers and Lis, 2015). The pervasive acetylation of H3K27 in H3.3K27M-bearing cell lines localizes to repetitive elements (REs), including endogenous retroviral sequences (ERV), which are usually tightly controlled and silenced in the genome (Krug et al., 2019). This H3K27ac leads to increased expression of ERV elements and ultimately to so-called viral mimicry in H3K27M pHGGs (Pathania et al., 2017; Krug et al., 2019).

## Molecular and Phenotypic Differences in pHGGs Expressing Either H3.1K27M or H3.3K27M

Distinct characteristics of pHGG have been found, depending on whether H3K27M mutation occurs in H3.1 or H3.3 histone variants. The differences in the associated secondary mutations are also apparent, as H3.3K27M mainly co-occurs with mutations of *TP53* (coding for tumor suppressor protein 53) and amplification of *PDGFRA* (encoding platelet derived growth factor alpha). In contrast, H3.1K27M co-occurs with mutations in *ACVR1* (encoding the activin receptor type-1) and genes involved in the PI3K (phosphatidylinositol 3-kinase)-signaling pathway (Wu et al., 2012; Buczkowicz et al., 2014; Fontebasso et al., 2014; Taylor et al., 2014; Mackay et al., 2017). Moreover, distinct genome-wide distribution patterns of these oncohistones were found, with H3.3K27M incorporating into the nucleosomes at the active chromatin sites and gene bodies, while H3.1K27M localizing uniformly across the genome. These changes reflect the expression and the genome-wide distribution pattern of wild-type H3.3 and H3.1 variants, respectively (Nagaraja et al., 2019). Moreover, tumors with either H3.1K27M or H3.3K27M variants have distinct profiles of active enhancers and promoters, potentially influencing the treatment response. For example, H3.3K27M-expressing tumors exhibit increased activity of enhancers regulating genes of the noncanonical WNT (Wingless/Integrated) signaling and cytoskeletal remodeling. In contrast, enhancers of H3.1K27M-expressing tumors control genes implicated in the PI3K and p38 MAPK (mitogen activated protein kinases) signaling pathways (Nagaraja et al., 2019). In accordance with the hypothesis that H3K27M has an inhibitory effect on the PRC2 complex, a detailed study showed that these two mutated H3 variants disrupt different PRC2 targets (Nagaraja et al., 2019). Finally, DNA methylation profiles can also distinguish H3.1K27M and H3.3K27M histone variant-expressing cells (Sturm et al., 2012; Castel et al., 2015, 2018).

## A Search for Specific Epigenetic Vulnerabilities of H3K27M pHGGs

### Targeting Modifiers of H3K27me3

**FIGURE 1:**
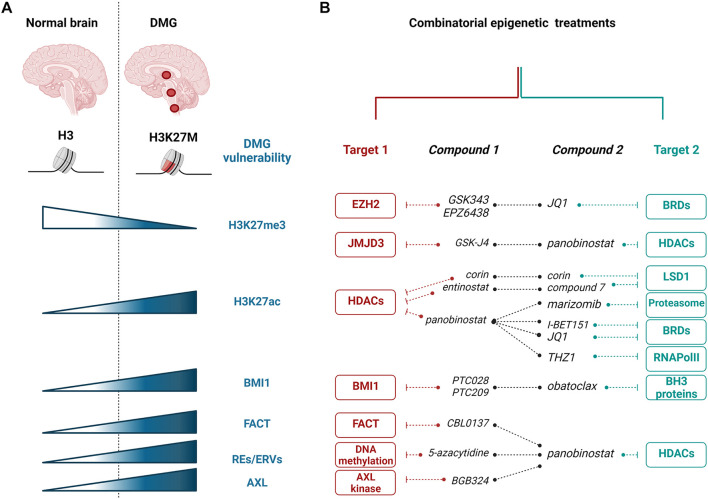
Epigenetic targeting of H3K27M diffuse midline gliomas (DMGs). **(A)** Most of DMGs (localizing to the thalamus, brainstem, or spinal cord) are characterized by H3K27M substitution. The mutation leading to H3K27M is responsible for numerous changes in DMG cells, including the decrease in H3K27me3 and the increase in H3K27ac histone modifications, and increased expression of BMI1, FACT, and REs/ERVs. **(B)** Epigenetic vulnerabilities of H3K27M DMGs were targeted with epigenetic enzyme inhibitors and showed promising effects in preclinical studies using in vitro and in vivo models. Red and teal colors showed various targets that are inhibited with specific compounds in combinatorial treatments, as indicated. EZH2, enhancer of zeste homolog 2; JMJD3, Jumonji domain-containing 3; HDACs, histone deacetylases; BRDs, bromodomain containing proteins; LSD1, lysine-specific demethylase 1; RNAPolII, RNA Polymerase II; BH3, Bcl-2 Homology 3; BMI1, B cell-specific Moloney murine leukemia virus integration site 1; FACT, facilitates chromatin transcription complex; REs, repetitive elements; ERVs, endogenous retroviral sequences; AXL, AXL receptor tyrosine kinase. The figure was created with BioRender.com.

A set of epigenetic alterations in H3K27M pHGGs created opportunities for therapeutic approaches (Figure 1A). One such strategy was to restore the H3K27me3 histone repressive mark. This strategy was achieved by selective inhibition of H3K27me3 demethylases (KDMs), namely KDM6 subfamily of Jumonji domain-containing 3 (JMJD3) K27 demethylases (Agger et al., 2007). Compound GSK-J4 specifically inhibited JMJD3, but not UTX (ubiquitously transcribed tetratricopeptide repeat on chromosome X, encoded by *KDM6A*), and decreased cell viability and clonogenic growth, while increasing the S-phase cell-cycle arrest and apoptosis (Hashizume et al., 2014). GSK-J4 was also potent in extending the survival of mice bearing H3K27M tumors. Importantly, the efficacy of GSK-J4 was more significant in mutant cells compared to H3 wild-type cells or the H3G34V-mutant cells. GSK-J4 treatment resulted in impaired DNA repair by homologous recombination and increased radiosensitivity of these cells (Katagi et al., 2019). At the moment, there are constrains of using GSK-J4 in clinical trials as it rapidly converts into an active drug GSK-J1 and has limited penetration into the brain.

While H3K27me3 levels are very low in H3K27M tumors, these marks are not entirely erased, and some loci in the genome retain this repressive mark (Chan et al., 2013). The residual methylation of the H3K27 residue is critical for DIPG survival and proliferation, as well as silencing of neural differentiation genes (Piunti et al., 2017). The PRC2 complex is required for the proliferative potential of H3K27M pHGGs, e.g., by silencing the expression of the tumor suppressor gene encoding p16^INK4A^, through a remaining H3K27me3 mark at its promoter (Mohammad et al., 2017). Consequently, targeting EZH2, the methyltransferase subunit of the PRC2 complex, effectively decreased cell viability, proliferation, and prolonged survival of mice bearing H3K27M tumors (Mohammad et al., 2017).

### Interfering With H3K27ac and Transcriptional Activity in H3K27M pHGGs

While H3K27M pHGGs have elevated levels of H3K27ac when compared to the tumors expressing the wild-type H3 variants, screening of various epigenetic modifying drugs showed that histone deacetylase (HDAC) inhibitors are among the most potent compounds decreasing the survival of pHGG cells (Grasso et al., 2015; Anastas et al., 2019). Several HDAC inhibitors were tested so far in pHGGs, including vorinostat, entinostat, and panobinostat (LBH589), with the latter showing the highest potency and being the most explored as a single agent or in combination with other drugs (see further sections) (Grasso et al., 2015; Nagaraja et al., 2017; Anastas et al., 2019; Krug et al., 2019; Lin et al., 2019; Ehteda et al., 2021; Vitanza et al., 2021). In addition, quisinostat and romidepsin emerged recently as potent HDAC inhibitors in treatment-naïve biopsy-derived DMG models (Vitanza et al., 2021). Overall, preclinical data showed that HDAC inhibitors potently kill DMG cells, reduce cell proliferation, and inhibit tumor growth *in vitro* and in animal models (Grasso et al., 2015; Anastas et al., 2019; Vitanza et al., 2021).

However, studies of panobinostat effects showed that despite a high potency of this drug at subnanomolar concentrations and an immediate impact on cell viability, the acquired resistance of H3K27M cells appears after the prolonged treatment (Grasso et al., 2015). Moreover, the systemic administration of panobinostat at different schedules and well-tolerated doses in mouse DIPG models and xenografts did not bring survival benefits compared to the controls (Hennika et al., 2017). Alternative compounds or combinations with other drugs might be needed to overcome treatment resistance. Moreover, a thorough investigation of the panobinostat action and the mechanism of cellular response is required. So far, it has been shown that, as expected, panobinostat increased histone acetylation. In addition, panobinostat partially restored the levels of H3K27me3 and normalized the pattern of gene expression affected by H3K27M (Grasso et al., 2015). The mechanism of the H3K27me3 restoration by HDAC inhibition is unclear. However, it was shown in a separate study that poly-acetylated H3 tails are able to “detoxify” a PRC2 complex by restoring its activity in H3K27M tumors (Brown et al., 2014). Moreover, the H3K27me3 increase in response to HDAC inhibition was observed in other tumor types not expressing H3K27M (Halsall et al., 2015). In the context of induced levels of H3K27ac at the REs and increased expression of ERVs in H3K27M pHGGs, the use of panobinostat increased histone acetylation at these sites and contributed to the so-called viral mimicry. Of note, mass spectrometry analysis showed that the increase in H3K27ac in response to panobinostat occurs mainly at H3.3 and not H3.1 or H3.2 histone variants (Krug et al., 2019).

Acetylation of the H3 N-tail, including H3K27ac, recruits bromodomain and extraterminal (BET) proteins, including BRD2 (Bromodomain Containing 2) or BRD4 (Bromodomain Containing 4), which are epigenetic readers of histone acetylation and, in turn, recruit cofactors for transcription initiation and ultimately trigger activation of RNA PolII-dependent transcription (Jonkers and Lis, 2015; Fujisawa and Filippakopoulos, 2017). Since H3K27ac is elevated in H3K27M tumors, targeting the BET proteins brings another intuitive strategy against H3K27M pHGGs. Piunti and colleagues showed that when an H3K27M oncohistone forms heterotypic nucleosomes with H3K27ac, these complexes recruit BRD2 and BRD4 to the chromatin (Piunti et al., 2017). Subsequently, targeting these cells with BET inhibitors JQ1 or I-BET151 significantly reduced cell viability and decreased overall H3K27ac levels. In addition, JQ1 and I-BET151 also potently inhibited tumor growth, decreased cell proliferation, and induced differentiation, as evidenced by increased expression of differentiation genes, including *TUBB3*, in mouse xenograft models. Interestingly, the use of JQ1 in xenografts *in vivo* was more efficient than using the GSK-J4 inhibitor against JMJD3 H3K27 demethylases (Piunti et al., 2017). However, it was not clarified whether JQ1 exhibited stronger toxicity in H3K27M-expressing cells when compared to H3 wild-type cells as did GSK-J4 (Hashizume et al., 2014). The potential of using JQ1 against DIPG tumors was also demonstrated by other studies, in parallel to targeting RNA PolII phosphorylation with the CDK7 (cyclin-dependent kinase 7) inhibitor, THZ1 (Taylor et al., 2015; Nagaraja et al., 2017).

Another study independently exposed a transcriptional activity as a vulnerability of DIPG tumors. Screening using RNAi library identified AF4/FMR2 Family Member 4 (AFF4), a scaffold protein in the super elongation complex (SEC), as a critical protein in maintaining the clonogenic potential, promoting self-renewal potential and stemness of DIPG tumors (Dahl et al., 2020). Depletion of AFF4 with shRNA induced the expression of prodifferentiation genes and reduced the self-renewal of DIPG cells. Subsequently, the authors used the CDK9 inhibitors atuveciclib and AZD4573, which interfere with the function of SEC by blocking the release of RNA Pol II from promoter-proximal pausing, which is normally mediated by RNA Pol II phosphorylation by CDK9 (Peterlin and Price, 2006; Zhou et al., 2012). Similarly to depletion of AFF4, CDK9 inhibitors induced the expression of pro-differentiation genes and reduced the self-renewal capacity of DIPG cells. In addition, atuveciclib and AZD4573 showed therapeutic benefits by delaying tumor growth and increasing mice survival in the orthotopic xenograft models of DMG (Dahl et al., 2020).

## Combinatorial Epigenetic Treatments

### Targeting HDACs and Histone Demethylases

While hopes regarding high *in vitro* potency of panobinostat in targeting H3K27M pHGGs were diminished by *in vivo* data showing a negligible survival effect at well-tolerable doses in animal tumor models, subsequent strategies evolved to combine the inhibition of HDACs with other epigenetic targets (Figure 1B; Grasso et al., 2015; Hennika et al., 2017). As a first attempt, a synergy in using panobinostat with GSK-J4 was shown. This treatment required much lower doses of each drug in combination than as single agents (Grasso et al., 2015). However, little is known about the exact molecular changes induced by this combinatorial treatment.

In another study, Anastas et al. (2019) searched for the drugs that would sensitize H3K27M-expressing cells to HDAC inhibitors. Cells were subjected to CRISPR KO screening with sgRNA library against 1,354 candidate genes related to chromatin regulation and treated with panobinostat for 3 weeks (Anastas et al., 2019). Among the top hits, *KDM1A*, encoding H3K4me1/2-specific demethylase lysine-specific demethylase 1 (LSD1), came out as a promising candidate (Anastas et al., 2019). Subsequently, the authors showed that simultaneous targeting of HDACs and LSD1 in H3K27M cells with entinostat and compound 7, respectively, synergistically decreased cell survival and proliferation. As an attractive alternative, the authors used a bifunctional single-molecule inhibitor, Corin, derived from compound 7 and entinostat, to inhibit both HDACs and LSD1 (Anastas et al., 2019). While the LSD1 inhibitor alone did not affect the cell viability of H3K27M cells, the use of Corin or a combination of entinostat with the LSD1 inhibitor compound 7 enhanced the sensitivity of these cells compared to the HDAC inhibitor alone. *In vivo*, Corin significantly reduced the xenograft growth when delivered intracranially as convection-enhanced delivery (CED). The rescue of H3K27ac and H3K27me3 was visible in the areas surrounding Corin injections but not in more distant tumor regions. Corin induced the differentiation of H3K27M cells and altered gene expression profiles differently than treatment with entinostat and compound 7 alone (Anastas et al., 2019). Interestingly, by performing gene set enrichment analysis on normal brains and DIPG tumor expression datasets, the authors found a strong analogy between the Corin-dependent gene expression signature and the one in a normal brain. This similarity emphasized the role of Corin in DIPG differentiation and reversing the gene expression pattern in tumor cells (Anastas et al., 2019).

### Targeting HDACs and Transcription

Since the initial potency of panobinostat as a single agent was strong in DIPG preclinical studies, there was a need to find ways to overcome the resistance of cells to prolonged treatments with this drug (Grasso et al., 2015). Nagaraja et al. (2017) showed that targeting the transcriptional activity of DIPG cells through the CDK7 inhibitor THZ1 sensitized panobinostat-resistant DIPG cells. However, this effect was not achieved when panobinostat was combined with JQ1, a BET protein inhibitor. This might be explained by the fact that panobinostat and JQ1 induce similar transcriptional programs, while THZ1 treatment leads to a distinct transcriptional signature and thus inhibiting panobinostat-resistant cells with THZ1 has additional benefits (Nagaraja et al., 2017).

### Targeting HDACs and DNA Methylation

Krug et al. (2019) showed that H3K27M pHGGs exhibit increased pervasive H3K27ac mark at REs, including ERVs, resulting in higher expression of these sequences. In healthy tissues, the expression of REs is tightly controlled and silenced in the genome via multiple epigenetic mechanisms, including DNA methylation or deposition of histone repressive marks (H3K9me3, H4K20me3, and H3K27me3) (Walsh et al., 1998; Bestor and Bourc’his, 2004). Hence, the authors speculated that demethylation of DNA with 5-azacytidine and additional increases in H3K27ac with panobinostat might increase the expression of REs even further and affect the viability of these cells (Krug et al., 2019). Indeed, the combination of panobinostat with 5-azacytidine significantly improved the survival of mice bearing H3K27M tumors when compared to either drug alone. The effect of 5-azacytidine alone or in combination with panobinostat was more substantial in H3K27M tumors when compared to the H3 wild-type group (Krug et al., 2019).

### Targeting HDACs and FACT Complex

A recent study showed that subunits of the FACT (facilitates chromatin transcription) complex, namely structure-specific recognition protein 1 (SSRP1) and suppressor of Ty16 (SPT16), are overexpressed in DIPG tumors when compared to normal brain tissues and normal human astrocytes (Ehteda et al., 2021). The FACT complex works as a histone chaperone interacting with nucleosomes and is involved in DNA repair, DNA replication, and transcription (Hsieh et al., 2013; Prendergast et al., 2020). Ehteda et al. (2021) showed that the SSRP1 subunit of FACT directly interacts with the H3.3K27M mutant histone. The FACT inhibitor, CBL0137, significantly decreased the survival of DIPG cells and impaired the growth of xenografts in mice as a single agent and synergized with panobinostat to cause these effects. In addition, CBL0137 increased trimethylation and acetylation of lysine 27 at H3, although the mechanism for this effect has not been explained (Ehteda et al., 2021). Moreover, the decrease in tumor sphere survival by CBL0137 was more pronounced in H3K27M-expressing DIPG spheres than in H3 wild-type human fibroblasts, normal human astrocytes, and H3K27 wild-type DIPG cells (Ehteda et al., 2021). Overall, the FACT complex emerged as a promising target specifically in H3K27M-expressing pHGGs.

### Targeting HDACs and Proteasome

A high-throughput screen using a collection of approved drugs tested the efficiency of 9,195 drug–drug combinations in order to find agents that synergize in targeting pHGG cells (Lin et al., 2019). A synergy between panobinostat and a proteasome inhibitor, marizomib, in increasing cellular toxicity was found across the DIPG cell lines expressing either H3.1 or H3.3 K27M mutated histones as well as in patient-derived xenografts. However, an effect in decreasing cell survival and proliferation was equally strong in cells expressing either mutant or wild-type histone H3. The combination of panobinostat with marizomib induced profound transcriptional changes in pHGG cells, particularly a downregulation of cellular metabolism and respiration, as well as induction of unfolded protein response. The cytotoxic effect of the combined treatment was due to pushing cells into a metabolic catastrophe, which was then reversed by exogenously normalizing NAD^+^ cellular levels (Lin et al., 2019).

### Targeting HDACs and AXL

Ectopic expression of the H3K27M oncohistone in the murine embryonic hindbrain neural stem cells leads to the increased gene signature of epithelial to mesenchymal transition (EMT), which is also observed in DIPG tumors (Puget et al., 2012; Castel et al., 2015; Meel et al., 2020). An elevated expression of the AXL kinase, one of the initiators of the EMT, was identified in the DIPG biopsy samples and was correlated with the presence of H3K27M (Meel et al., 2020). This evidence provided a rationale for testing the AXL-specific inhibitor, BGB324, in DIPG cells. BGB324 significantly decreased cell viability at submicromolar concentrations. Moreover, inhibition of AXL with BGB324 or depletion with AXL-specific shRNA significantly impaired the invasion of neurospheres in the 3D matrigel invasion assay. AXL inhibition increased the expression of epithelial differentiation markers and downregulated the mesenchymal genes (Meel et al., 2020). Subsequently, AXL was tested in combination with panobinostat, as panobinostat alone was also shown to decrease the expression of mesenchymal genes (di Fazio et al., 2012; Rhodes et al., 2014; Grasso et al., 2015). The combination of BGB324 and panobinostat synergized in reducing cell viability in cells expressing H3K27M but not in cells with wild-type H3 histone variants. Importantly, this effect of treatment specificity to cells expressing the H3K27M oncohistone was not observed for panobinostat or BGB324 alone. The synergy between AXL and HDAC inhibition in antiproliferative effects in DIPG cells was also demonstrated with additional HDAC inhibitors and with shRNA against AXL, emphasizing the specificity of these drugs rather than potential off-target effects. Moreover, the combination of the AXL inhibitor and panobinostat synergized in decreasing cell migration and invasion, as well as in reversing the mesenchymal phenotype of DIPG cells, as shown by a decrease in the expression of genes such as *ZEB1*, *ZEB2, SNAI2, SOX2*, or *NES* and the induction of proneural pathways. While panobinostat alone sensitized DIPG cells to radiotherapy, this was enhanced by the addition of BGB324, as shown by neurosphere formation assay. Combined radiosensitization effect of panobinostat with BGB324 was justified by the most robust repression of DNA repair genes, including *FANCD2* and *RAD51*. In parallel to a decrease in DNA repair genes, an increase in γH2AX, a marker of DNA damage, was strongest in the combination treatments (Meel et al., 2020). Finally, it was demonstrated that BGB324 has a capacity of crossing the blood–brain barrier after oral administration. Moreover, when combined with panobinostat delivered through CED, BGB324 has a synergistic effect in delaying the tumor growth in mouse models of DIPG (Meel et al., 2020).

### Targeting BET Bromodomains and PRC2

While single-agent targeting against either EZH2 or BET family of proteins in DIPG tumors proved to be efficient (Mohammad et al., 2017; Piunti et al., 2017), one study combined these two strategies by using the EZH2 inhibitor EPZ6438 and the JQ1 inhibitor against the BET proteins (Zhang et al., 2017). The authors used mouse primary neural stem cell (NSC) cultures, in which HA-PDGFB and either flag-H3.3K27M or flag-H3.3K27 wild-type transgenes were overexpressed. When injected into the mouse pons, H3.3 K27M NSCs formed larger tumors than those expressing wild-type histone H3. An even stronger effect of the combined JQ1 and EPZ6438 treatment in inhibiting tumor growth was observed compared to the single agents. The more substantial impact of this drug combination was explained by a decrease in H3K27me3 levels at the tumor suppressor gene p16^INK4A^ (Zhang et al., 2017). Interestingly, another study showed a synergistic effect of combining the inhibition of EZH2 and HDACs with the induction of TRAIL-dependent cell death (by combining drugs such as EPZ6438, panobinostat, and ONC201/TIC10, respectively) (Zhang et al., 2021). While this approach showed promising results in inducing cell death *in vitro* in DIPG cells, it should be further verified in preclinical animal models.

### Targeting BET Bromodomains and CBP

The BET bromodomain inhibitor JQ1 was tested in combination with a structural inhibitor of the acetyltransferase, CBP (CREB binding protein). ICG-001, the CBP inhibitor, prevents the interaction of CBP with other proteins, rather than interfering with its catalytic function (Wiese et al., 2020). Both inhibitors, when compared alone as single agents, had a potential in decreasing protumorigenic functions of DIPG cells, such as reducing cellular survival, sphere formation, migration, invasion, or radioresistance. Interestingly, these drugs had opposing effects on the regulation of the majority of super-enhancers in DIPG cells when used separately: JQ1 inhibited and ICG-001 activated these regulatory sites. Moreover, JQ1 and ICG-001 alone inadvertently activated a set of super-enhancers, which was reversed when these drugs were used in combination (Wiese et al., 2020). Therefore, the cytotoxic and anti-self-renewing effects of these drugs when used in combination were very strong and give a rationale for further preclinical testing in animal models and potentially in clinical trials.

### Targeting BMI1 and Anti-apoptotic Pathway

Another genetic screen searching for DIPG vulnerabilities found BMI1 (B cell-specific Moloney murine leukemia virus integration site 1), a component of the PRC1 complex, as a potential target in DIPG tumors (Balakrishnan et al., 2020). The PRC1 complex is a chromatin remodeler that monoubiquitinates lysine 119 at histone H2A (H2AK119Ub) (Barbour et al., 2020). Balakrishnan et al. (2020) found that BMI1 and H2AK119Ub histone mark are increased in H3K27M-expressing tumors compared to the normal pons. Increased expression of BMI1 was directly driven by the presence of H3K27M. Subsequently, inhibition of BMI1 with PTC209 or PTC028 inhibitors significantly inhibited the proliferation of tumor cells. A higher sensitivity was observed in cells expressing H3K27M mutation when compared to H3K27M-CRISPR-deleted cells (Kumar et al., 2017; Balakrishnan et al., 2020). Phenotypically, inhibition of BMI1 impaired stem cell renewal in DIPG cells by reversing the BMI1 and H2A-K119Ub-mediated repression of differentiation-related genes. In addition, BMI1 inhibition repressed DIPG cell proliferation and induced senescence concomitant with upregulated expression of p16 and p21 tumor suppressor genes. However, senescence induced by BMI1 inhibition led to an activation of the senescence-associated secretory phenotype (SASP), which increases the risk of a future recurrence of tumors. To overcome this hurdle, the authors combined the BMI1 inhibitor together with obatoclax, a BH3 mimetic, which binds to the anti-apoptotic BCL2 family of proteins and induces apoptosis in cells. Combined treatment with PTC028 and obatoclax had the strongest effect in decreasing tumor growth and improving mouse survival compared to single drugs. In summary, this study highlighted BMI1 as a therapeutic target that is specifically upregulated in the presence of H3K27M substitution. It provided a strong rationale to target the BMI1 chromatin remodeler together with the BH3 mimetic to inhibit the growth of H3K27M pHGGs and prevent SASP reprogramming (Balakrishnan et al., 2020).

## Clinical Trials Including Targeting Epigenetic Modifiers in pHGGs

Multiple clinical trials including the use of drugs targeting the epigenetic landscape of pHGGs had been established to date (Table 1). HDAC inhibitors such as vorinostat and panobinostat have been clinically studied with favorable safety profiles in children. However, there have been concerns regarding the efficiency of panobinostat at crossing the blood–brain barrier (Rasmussen et al., 2015). A related clinical trial has used panobinostat in children with DIPG to study the side effects and the most effective dose in recurrent and progressing DIPG (NCT02717455). Another trial has used an HDAC/PI3K inhibitor fimepinostat in patients with DIPG, HGG, or medulloblastoma (NCT03893487). An ongoing clinical trial has combined vorinostat and temsirolimus (an mTOR inhibitor), in experiment arms with and without radiotherapy (NCT02420613). Another clinical trial in DIPG includes a testing BMI1 inhibitor, PTC596 (NCT03605550). This trial is particularly promising, as a recent report showed antitumor effects with other BMI1 inhibitors, PTC209 or PTC028 (Balakrishnan et al., 2020). An ongoing Phase I/II clinical trial tries to establish the safety of a synthetic peptide vaccine specific for the H3.3K27M epitope in combination with poly-ICLC and the PD-1 inhibitor, Nivolumab, in newly diagnosed DIPG patients and other gliomas that are tested positive for H3.3K27M (NCT02960230).

**TABLE 1:** A summary of past and present clinical trials in pHGGs, which include epigenetic treatments.

ID	Clinical trial	Epigenetic target	Compound
NCT02899715	Panobinostat in treating younger patients with progressive diffuse intrinsic pontine glioma	HDAC	Panobinostat
NCT02717455	Trial of panobinostat in children with diffuse intrinsic pontine glioma	HDAC	Panobinostat
NCT03566199	MTX110 by convection-enhanced delivery in treating participants with newly diagnosed diffuse intrinsic pontine glioma	HDAC	MTX110 (panobinostat nanoparticle formulation MTX110)
NCT01189266	Vorinostat and radiation therapy followed by maintenance therapy with vorinostat in treating younger patients with newly diagnosed diffuse intrinsic pontine glioma	HDAC	Vorinostat
NCT02420613	Vorinostat and temsirolimus with or without radiation therapy in treating younger patients with newly diagnosed or progressive diffuse intrinsic pontine glioma	HDAC and mTOR	Vorinostat, Temsirolimus
NCT00879437	Valproic acid and radiation followed by maintenance valproic acid and bevacizumab in children with high-grade gliomas or diffuse intrinsic pontine glioma	HDAC	Valproic acid
NCT03893487	Fimepinostat in treating brain tumors in children and young adults	HDAC and PI3K	Fimepinostat
NCT03605550	A Phase 1b study of PTC596 in children with newly diagnosed diffuse intrinsic pontine glioma and high-grade glioma	BMI1	PTC596
NCT02960230	H3.3K27M peptide vaccine with nivolumab for children with newly diagnosed DIPG and other gliomas	H3K27M epitope and PD-1	H3.3K27M Peptide, Nivolumab

The blood–brain barrier is a major challenge that needs to be addressed for any epigenetic drugs to achieve therapeutic efficacy in DIPG therapy. To circumvent this issue, CED technique is being explored as an alternative where the drugs are directly delivered to the tumor site with limited side effects (Souweidane et al., 2018). There are open clinical trials for the delivery of panobinostat with gadoteridol using CED in patients newly diagnosed with DIPG (NCT03566199). A lot of complex issues also surround the usage of CED in clinical trials. For example, targeted drugs such as tyrosine kinase inhibitors are promising agents; however, they are not soluble and currently do not have an IV/aqueous formulation to allow their delivery using CED. Along with this, there are also legal and logistical issues that hinder the scalability of complicated therapeutics such as CED (Wierzbicki et al., 2020).

## Summary and Future Perspectives

While radiotherapy is the current standard treatment regimen for H3K27M-positive pHGGs, the unique biology of these tumors opens avenues for promising epigenetic therapies. The hallmarks of these tumors, such as loss of H3K27me3, increased H3K27ac, or changes in the expression of chromatin modifiers, e.g., BMI1, invite further research into the efficacy and molecular response to these treatments. Inhibitors of H3K27me3 demethylases, DNA methylation, BMI1, or FACT show strong potency in targeting H3K27M-containing tumors when compared to pHGG cells expressing wild-type H3, suggesting potentially smaller systemic toxicity in patients. However, further research is needed to develop novel compounds with a similar function that will exhibit higher stability *in vivo* and cross the blood–brain barrier. Of the drugs summarized in this review, some have been shown to cross the blood–brain barrier after systemic administration at least in the preclinical animal models, including marizomib, BGB324, JQ1, THZ1, obatoclax, CBL0137, or Azacytidine (Korb et al., 2015; Lin et al., 2019; Balakrishnan et al., 2020; Butler et al., 2020; Meel et al., 2020; Ehteda et al., 2021). Panobinostat and Corin reached sufficient levels in the brain tumors when delivered via CED (Grasso et al., 2015; Singleton et al., 2018; Anastas et al., 2019; Meel et al., 2020). For the future perspective, the effects of promising combinatorial epigenetic treatments should be tested for their radiosensitizing capabilities to verify the rationale for including these combinations in the clinical trials with standard radiotherapy in pHGGs. For example, the radiosensitizing effects of panobinostat, BGB324, or GSK-J4 had been demonstrated (Katagi et al., 2019; Meel et al., 2020). However, similar tests on other combinatorial approaches in pHGGs would be informative for the potential future clinical trials. Recently, it was demonstrated that the radiosensitizing effect of GSK-J4 was further enhanced by combinatorial treatment with APR-246, an agent that targets p53 mutant proteins and leads to the accumulation of reactive oxygen species (Bykov et al., 2018; Nikolaev et al., 2020).

The promising efficacy of recent combinatorial epigenetic treatments in pHGGs invites novel drug combinations, some of which may include small molecule inhibitors against the cell surface receptors or druggable kinases, e.g., *PDGFR* and CDK4/6 (Hoeman et al., 2018). Another direction for combinatorial treatments explores mutations that coexist with the H3K27M substitution in pHGGs. As an example, mutations in the *PPM1D* gene were identified in approximately 9–23% of DIPG samples and mostly co-occur with the H3K27M oncohistone (Taylor et al., 2014; Wu et al., 2014; Zhang et al., 2014; El Ayoubi et al., 2017). *PPM1D* encodes a phosphatase responsible for dephosphorylation of multiple proteins involved in DNA damage response and DNA repair pathways (Deng et al., 2020). A recent study showed that the inhibitor of PPM1D, GSK2830371, sensitized DIPG cells with the background of *PPM1D* mutation toward PARP inhibition, emphasizing the DNA repair pathways as a vulnerability of these tumors (Wang et al., 2020).

It is important to note that while numerous studies have tried to determine the precise effects of tested epigenetic drugs, it is almost impossible to explore all the potential consequences of epigenetic inhibitors due to pleiotropic effects exerted by the targeted enzymes and induction of widespread global changes in the chromatin. The typical phenotype induced by many of the successful inhibitors included induction of differentiation of H3K27M glioma cells and reduction of the self-renewing potential, as observed for HDAC, HDAC/LSD1, BET bromodomain, CDK9, or BMI1 inhibitors (Nagaraja et al., 2017; Piunti et al., 2017; Anastas et al., 2019; Balakrishnan et al., 2020; Dahl et al., 2020; Meel et al., 2020). Broadly available next-generation sequencing techniques were key in determining the genome-wide changes in gene expression, chromatin accessibility, or localization of specific histone modifications, which gave a broad view on the global changes induced by tested epigenetic treatments. For example, identification of pervasive H3K27ac at the REs and ERVs in the H3K27M-expressing tumors exposed a specific vulnerability of these tumors that was further enhanced by the use of global inhibitors toward HDACs and DNA methylation (Krug et al., 2019).

Finally, little is known on the effects of epigenetic therapies on the tumor microenvironment of pediatric brain tumors, including the immune system. Latest advances in single-cell transcriptomics and epigenomics will be essential in providing new insights into the right selection of drugs and targets in the context of tumor immune responses. The common efforts of scientists and neuro-oncologists will be critical in translating these promising preclinical data into clinical trials.

## Author Contributions

KL and JM conceived the hypothesis. KL and CJ did the literature search. KL, CJ, BK, and JM co-wrote the manuscript. All authors contributed to the article and approved the submitted version.

## Conflict of Interest

The authors declare that the research was conducted in the absence of any commercial or financial relationships that could be construed as a potential conflict of interest.

## Publisher’s Note

All claims expressed in this article are solely those of the authors and do not necessarily represent those of their affiliated organizations, or those of the publisher, the editors and the reviewers. Any product that may be evaluated in this article, or claim that may be made by its manufacturer, is not guaranteed or endorsed by the publisher.
